# Changes in Internet use patterns among older adults in England from before to after the outbreak of the COVID-19 pandemic

**DOI:** 10.1038/s41598-023-30882-8

**Published:** 2023-03-09

**Authors:** Claryn S. J. Kung, Andrew Steptoe

**Affiliations:** 1 Department of Epidemiology and Public Health, University College London, 1-19 Torrington Place, London, WC1E 7HB UK; 2 Department of Behavioural Science and Health, University College London, 1-19 Torrington Place, London, WC1E 7HB UK

**Keywords:** Geriatrics, Epidemiology

## Abstract

The COVID-19 pandemic brought about an increased reliance on the Internet for various daily activities. Given the known digital divide, it is important to understand whether older adults changed their Internet use patterns, but current evidence is limited to cross-sectional studies. This study documents changes in frequency and types of Internet use among older adults from before to shortly after the outbreak of the COVID-19 pandemic (2018/2019 to June/July 2020), and the factors predicting regular use during these early days of the pandemic. Using data on 6,840 adults aged 50 + from the nationally representative English Longitudinal Study of Ageing, we apply longitudinal fixed-effects models to examine within-individual changes in Internet use behaviour. There was no change in the likelihood of daily Internet use between 2018/2019 and June/July 2020, despite the increased digitalisation of services over the pandemic. Daily use in June/July 2020 was negatively related to age, neighbourhood deprivation, and loneliness, and positively related to partnership status, education, employment, income, and organisation membership. Using the Internet for making calls and getting information about Government services increased, which was important given the social restrictions and overall uncertainty. However, Internet use for finding health-related information decreased. As the world moves towards digital alternatives post-pandemic, it is important to continually ensure older adults are not at risk of exclusion.

## Introduction

The COVID-19 pandemic has had a significant impact on the lives and wellbeing of populations globally^1–3^. Due to lockdown, social distancing, and stay-at-home restrictions, many individuals had to rely on the Internet for a diverse range of day-to-day functions, including working, communicating with family and friends, shopping, searching for information, and engaging with health services. Given the lower frequency of Internet use among older populations compared with younger cohorts (in a persistent ‘digital divide’)^4^, there are concerns that they have been particularly disadvantaged via digital exclusion^5,6^, especially as healthcare and other service providers become increasingly available on digital platforms^7–10^. It is important that societies ensure their older citizens are digitally active, in order that they can engage in all aspects of society, “as the world moves steadily towards a digital future”^11^.

Evidence on Internet use among older adults during the pandemic is still sparse. While some countries have reported a substantial increase in the number of older adult users (e.g., China)^12^, this has not been the case elsewhere: in England, older adults who rarely or never used the Internet before the outbreak, did not increase their use in the first three months of the pandemic^13^. Several cross-sectional studies have examined the types of Internet use among older adults and demographic predictors of these uses, but using data collected only after the onset of the pandemic^14–17^. These studies documented whether and how older adults use the Internet, including searching and sharing COVID-19-related information online, communicating, shopping, using social networking services, and banking, as well as differences between demographic subgroups in these types of uses. However, in the absence of pre-pandemic data, it is not possible to conclude whether these patterns of use have changed.

**Figure 1:**
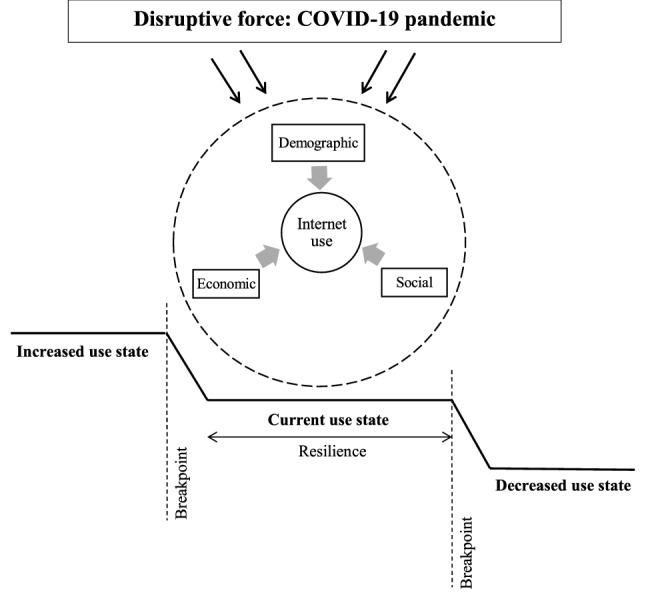
An adaptation of the Dynamics in Technology Use by Seniors (DITUS) framework. The DITUS framework^18^ was developed to explain technology use by independent-living seniors over time. The framework posits a core of interrelated factors linked to technology use, as well as disruptive forces that can induce changes to other levels of use. In line with these concepts, the primary research questions in this study are, (1) were there changes in Internet use patterns among older adults after the outbreak of the COVID-19 pandemic, and (2) what were the factors predicting Internet use after the outbreak of the pandemic?

In this study, we first provide evidence on whether, following the onset of the COVID-19 pandemic, there were changes in the frequency and types of Internet use among older adults, compared with pre-pandemic levels. Second, we describe the pre-pandemic demographic, economic, and social factors linked to Internet use after the outbreak of the pandemic. Figure 1 organises these two research aims using the Dynamics in Technology Use by Seniors (DITUS) framework^18^, which is a conceptual model that explains the changes and stability in technology use by independent-living seniors over time. The framework summarises not only the factors that are closely linked to use (e.g., need compatibility, proficiency to use, external support), but also the forces that could induce changes in use (e.g., changes in personal needs, financial conditions, physical environment; life events; competition with alternative means). These disruptive forces influence the factors, and their resulting dynamics can shift use to an increased or decreased state, when disruptions are sufficiently intense to achieve breakpoints.

We interrogate the English Longitudinal Study of Ageing (ELSA), a nationally representative of sample of adults aged 50 + in England. Specifically, we use data from its 2018/2019 (pre-COVID-19) and June/July 2020 waves (shortly after the outbreak of the pandemic). With respect to the first research aim, the pandemic and its ensuing restrictions may have indeed increased older adults’ need to use the Internet for various purposes, including communication and engagement with services, given limitations of alternative means (e.g., face-to-face meetings with friends and family, in-person appointments at clinics, banks, and other establishments). On the other hand, certain robust barriers to Internet use faced by older adults, such as lack of familiarity and lack of technological literacy and skills^19^, lack of interest or trust in technologies^20^, inability to afford Internet access or relevant equipment, reduced access due to poor health or cognitive functioning^21,22^, geographical restrictions^23^, and technostress^24^, may have rendered their Internet use levels resilient to change during the pandemic. All things considered, we hypothesise that older adults did not change in their likelihood of regular use from before to shortly after the outbreak of the pandemic, although types of use would have changed due to the circumstances of the pandemic.

The second research aim adds to evidence from previous cross-sectional COVID-19 studies on concurrent factors predicting Internet use. Our longitudinal dataset allows us to examine how demographic, economic, and social factors measured prior to the pandemic, related to Internet use observed shortly after the outbreak of the pandemic, thus mitigating reverse causality concerns. Moreover, we are able to adjust for pre-pandemic Internet use, which would include all pre-pandemic observable and unobservable factors related to Internet use more generally. While the examples provided in the DITUS framework refer to more personal factors, the factors we consider are informed by the larger literature on Internet use among older adults. For instance, studies have found significant differences in their Internet use behaviours by gender, age, and ethnic minority status, as well as education, income, health, and social contacts (particularly contacts who are users themselves)^4,14,22,25–37^. These observable factors are nonetheless related to personal factors discussed in the DITUS framework: as examples, age, education, and income are related to proficiency to use^38^, whereas poor health and lack of experience (i.e., low levels of past Internet use) are related to technostress^24^ or negative emotions associated with Internet use. We hypothesise that even after controlling for pre-pandemic Internet use, at least some of these key factors would still be related to Internet use during the pandemic, given they are also related to personal experiences during the pandemic.

## Methods

### Study design and participants

The English Longitudinal Study of Ageing (ELSA) is an ongoing panel study that is nationally representative of adults aged 50 + and their partners, who reside in private households in England. The Study began in 2002 (Wave 1), with responses from 12,099 individuals from 7,934 households. Every two years, sample participants are interviewed on various dimensions of health, social, cognitive, and economic wellbeing; in addition, every four years, nurse visits are conducted for the collection of biological samples and anthropometric measurements. The most recent sweep of the study is Wave 9, with data collection spanning June 2018 and July 2019. The sample has also been refreshed at Waves 3, 4, 6, 7, and 9, to ensure the sample remains nationally representative^45^.

We also use Wave 1 of the ELSA COVID-19 Substudy, administered between June 3 and July 26, 2020. The survey was issued to 9,392 eligible members in 6,173 households, with 7,040 interviews completed (75% response rate). The survey was administered online (83%) or by telephone interview for those who unable to respond online (17%). From the 7,040 completed interviews in the COVID-19 Substudy Wave 1, 6,840 interviews provided non-missing Internet use data (after applying cross-sectional weights provided by the ELSA team)^41^.

The English Longitudinal Study of Ageing received ethical approval from the South Central-Berkshire Research Ethics Committee (21/SC/0030, 22nd March 2021), and the COVID-19 Substudy was approved by the University College London Research Ethics Committee. Informed consent was obtained from all participants, and all analyses in this study is performed in accordance with Committee guidelines.

### Internet use

In the COVID-19 Substudy, participants were asked, “Since the coronavirus outbreak, on average, how often did you use the Internet or email?”, to which they could respond “more than once a day”, “every day, or almost every day”, “at least once a week (but not every day)”, “at least once a month (but not every week)”, “less than monthly”, or “never”. From Waves 6 (2012/2013) to 9 (2018/2019), participants were asked “On average, how often do you use the Internet or email?”, with response options differing slightly from the COVID-19 Substudy. For consistency, we dichotomise this frequency variable at every wave, to indicate daily Internet use (Supplementary Table S1).

In the COVID-19 Substudy, participants using the Internet at least monthly were further asked about types of use over the last three months: for emails; video or voice calls; information on health-related issues; finances; shopping; social networking sites; news, newspaper, or blog websites; streaming or downloading TV/radio, music, games, or ebooks; and information about Government services. The list of options provided in previous waves were slightly different, so we remove or collapse several categories for consistency with the COVID-19 Substudy version (Supplementary Table S2).

### Demographic, economic, and social variables

We analyse whether the following factors, measured prior to the pandemic, predicted Internet use. We consider gender, age group (50–64, 65–74, 75–84, 85 + years), ethnicity (whether non-white), household composition (whether partnered and whether living with children), and whether living in a rural area. To reflect economic conditions, we include educational attainment and employment status, and quintiles of household income, wealth, and neighbourhood deprivation (using the Index of Multiple Deprivation). We additionally consider health: whether participants reported a limiting long-term condition, and whether they reported depressive symptomology, the latter observed as having at least four of eight possible symptoms from the Center for Epidemiologic Studies Depression Scale.

For social factors we include loneliness, captured using the sum of responses on the three-item UCLA Loneliness Scale; positive and negative support (based on three and four support statements, respectively) received from partner, children, other immediate family, and friends, with final scores averaged across support statements and sources; weekly contact (e.g., meet up, phone, email, text messages) with children, family, and friends not living with them; and membership of an organisation, club, or society.

Summary statistics for our analytical sample, weighted to be nationally representative, are presented in Table 1. Further information on factors, including constituting items, scoring, and standardisation methods, are presented in Supplementary Table S3.

**Table 1:** Sample pre-COVID-19 characteristics, by reported Internet use in June/July 2020.

	Total sample	Less than daily Internet use	Daily Internet use
*Demographic*			
Gender: male	0.471	0.407	0.491
Age in 2020			
50–64	0.450	0.211	0.527
65–74	0.301	0.282	0.308
75–84	0.179	0.318	0.134
85 +	0.069	0.189	0.030
Ethnicity: non-white	0.058	0.056	0.059
Partnered	0.706	0.543	0.759
Living with children	0.072	0.019	0.089
Government Office Region			
North East	0.053	0.067	0.049
North West	0.134	0.153	0.128
Yorkshire and The Humber	0.097	0.113	0.092
East Midlands	0.093	0.097	0.091
West Midlands	0.099	0.115	0.094
East of England	0.121	0.116	0.123
London	0.114	0.097	0.120
South East	0.171	0.137	0.182
South West	0.117	0.105	0.121
Live in rural area	0.240	0.210	0.250
Has a limiting long-term disability	0.310	0.471	0.258
Has depressive symptomatology^a^	0.117	0.180	0.097
*Economic*			
Educational attainment			
No qualification	0.157	0.351	0.094
Below O-level or other [NVQ1]	0.115	0.162	0.100
O-level [NVQ2]	0.229	0.216	0.233
A-level or higher education below degree [NVQ3]	0.272	0.212	0.292
Degree [NVQ4 and NVQ5]	0.227	0.059	0.281
Employment status			
Employed or self-employed	0.431	0.179	0.512
Retired	0.480	0.696	0.411
Other status (e.g., unemployed, sick)	0.088	0.125	0.076
Household income			
Quintile 1 (bottom, lowest)	0.191	0.322	0.149
Quintile 2	0.189	0.275	0.161
Quintile 3	0.193	0.202	0.191
Quintile 4	0.211	0.129	0.238
Quintile 5 (top, highest)	0.215	0.072	0.262
Wealth			
Quintile 1 (bottom, lowest)	0.216	0.326	0.181
Quintile 2	0.195	0.234	0.183
Quintile 3	0.193	0.204	0.190
Quintile 4	0.204	0.155	0.219
Quintile 5 (top, highest)	0.192	0.080	0.228
Index of Multiple Deprivation			
Quintile 1 (bottom, least deprived)	0.236	0.164	0.260
Quintile 2	0.241	0.194	0.256
Quintile 3	0.209	0.196	0.212
Quintile 4	0.181	0.230	0.165
Quintile 5 (top, most deprived)	0.133	0.215	0.106
*Social*			
UCLA Loneliness score^a^	1.365 (0.498)	1.483 (0.560)	1.327 (0.470)
Positive support^a^	2.162 (0.539)	2.153 (0.566)	2.165 (0.530)
Negative support^a^	0.630 (0.439)	0.624 (0.490)	0.632 (0.422)
At least weekly contact with non-household members^a^	0.023	0.027	0.022
Member of an organisation^a^	0.691	0.576	0.727
*N*	5,475	1,331	4,144

Figures are averages, weighted using cross-sectional weights provided by the ELSA team. Participants are only included if they provided a response to the item “Since the coronavirus outbreak, on average, how often did you use the internet or email?” in the COVID-19 Substudy. Figures in parentheses are standard deviations of continuous scores.

^a^Supplementary Table S3 presents further information on this factor, including constituting items, scoring, and standardisation methods.

### Statistical analysis

To produce representative estimates, we apply the relevant survey weights to all descriptive and statistical analyses^41^. All estimations are conducted using Stata 17.0. We first describe Internet use patterns over time, using the corresponding cross-sectional weights provided at each ELSA Wave, thereby adjusting for differences in the likelihood of participation among key subgroups.

Exploiting the longitudinal nature of the data, we examine within-individual changes in the likelihood of daily Internet use, using an individual fixed-effects model specification.

1 $$Y_{it} = W_{it} \gamma_{0} + W_{i,t - 1} \gamma_{1} + W_{i,t - 2} \gamma_{2} + Z_{i}^{^{\prime}} \alpha + X_{it}^{^{\prime}} \beta + \varepsilon_{it}$$

In Eq. (1), $Y_{it}$ denotes the likelihood of daily Internet use (dichotomising daily or more than once a day, vs. less frequent use) for individual *i* at time *t*, where *t* is the specific wave of interest (COVID-19 Substudy Wave 1, June/July 2020), and *t* − 1 and *t* − 2 represent ELSA Wave 9 (2018/2019) and Wave 8 (2016/2017), respectively. $W_{it}$ are wave dummy variables, each taking on the value of 1 if the observation is from the wave indicated, 0 otherwise. In this longitudinal specification, the estimate of interest is the dummy variable during the pandemic in June/July 2020 $\left( {W_{it} } \right)$, and we omit 2018/2019 $\left( {W_{i,t - 1} } \right)$ as the reference wave. The estimate for $\gamma_{0}$ therefore reflects the change in the likelihood of daily Internet use since the outbreak of the pandemic, relative to 2018/2019.

$Z_{i}^{^{\prime}}$ is a vector representing all observed and unobserved time-invariant covariates and is eliminated in all model estimations. For $X_{it}^{^{\prime}}$, which represents observed time-varying covariates, we include age, partnership and employment status, perceived financial difficulties, self-reported health, depressive symptomology, loneliness, levels of positive and negative support from partner, and contact with others. $\alpha$ and $\beta$ represent their corresponding coefficient vectors, and $\varepsilon_{it}$ is assumed to be a random error term. For all longitudinal analyses, we apply the ELSA longitudinal weights, which adjust the Wave 9 cross-sectional weights to account for differential non-response to the COVID-19 Substudy (Wave 1)^41^. Our weighted longitudinal analytic sample comprises 5,571 unique individuals, noting that the longitudinal weights effectively exclude partners of the ELSA core members.

To analyse the pre-COVID-19 factors predicting Internet use during the pandemic in June/July 2020, we employ a linear probability model specification as detailed below.

2 $$Y_{it} = \alpha + X_{i,t - 1}^{^{\prime}} \beta + \varepsilon_{i}$$

In Eq. (2), $Y_{it}$ denotes the likelihood of daily Internet use for individual *i* during the pandemic (*t* representing COVID-19 Substudy Wave 1, June/July 2020), whereas $X_{i,t - 1}^{^{\prime}}$ is a coefficient vector representing the array of factors measured before the outbreak (*t* − 1 representing Wave 9, 2018/2019). We also adjust for whether participants reported daily Internet use prior to the outbreak. In a sensitivity check we additionally examine the consistency of our conclusions when relying instead on an ordinal logistic regression specification, taking into account three frequency categories: daily Internet users (“more than once a day” or “every day, or almost every day”), irregular users (“at least once a week but not every day” or “at least once a month but not every week”), and non-users (“less than monthly” or “never”).

For ease of interpretation all factors are either dichotomised, such that each corresponding coefficient reflects the difference in likelihood of daily Internet use between the categories; or standardised, such that the coefficient reflects the difference in likelihood of daily use between every standard deviation difference in the factor. Here we apply the cross-sectional COVID-19 Substudy Wave 1 weights, resulting in a sample of 5,475 individuals in our complete-case analysis. Missing data on pre-pandemic predictors (cf. 6,840 with non-missing Internet use information in June/July 2020) are primarily due to non-completion of the ELSA Wave 9 self-completion questionnaire. Given that the self-completion questionnaire was administered by paper, non-completion was unlikely to depend on whether participants used the Internet regularly, suggesting that our complete-case analysis would not be biased^46^.

We also examine within-individual changes in the different types of Internet use, following Eq. (1): here $Y_{it}$ represents the likelihood of using the Internet for the corresponding type of use for individual *i* at time *t*. We restrict this analysis to participants reporting to use the Internet at least once a month (consistent with COVID-19 Substudy questionnaire filter). The coefficient of interest $\left( {\gamma_{0} } \right)$ therefore reflects the change in likelihood of reporting the corresponding type of use during the pandemic in June/July 2020, relative to 2018/2019. We additionally test whether the estimated change differs by key demographic and socioeconomic factors, by first splitting the sample by the corresponding factor, then running the regressions separately for each subsample, and finally testing for differences in the resulting estimates.

## Results

### Daily Internet use

**Figure 2:**
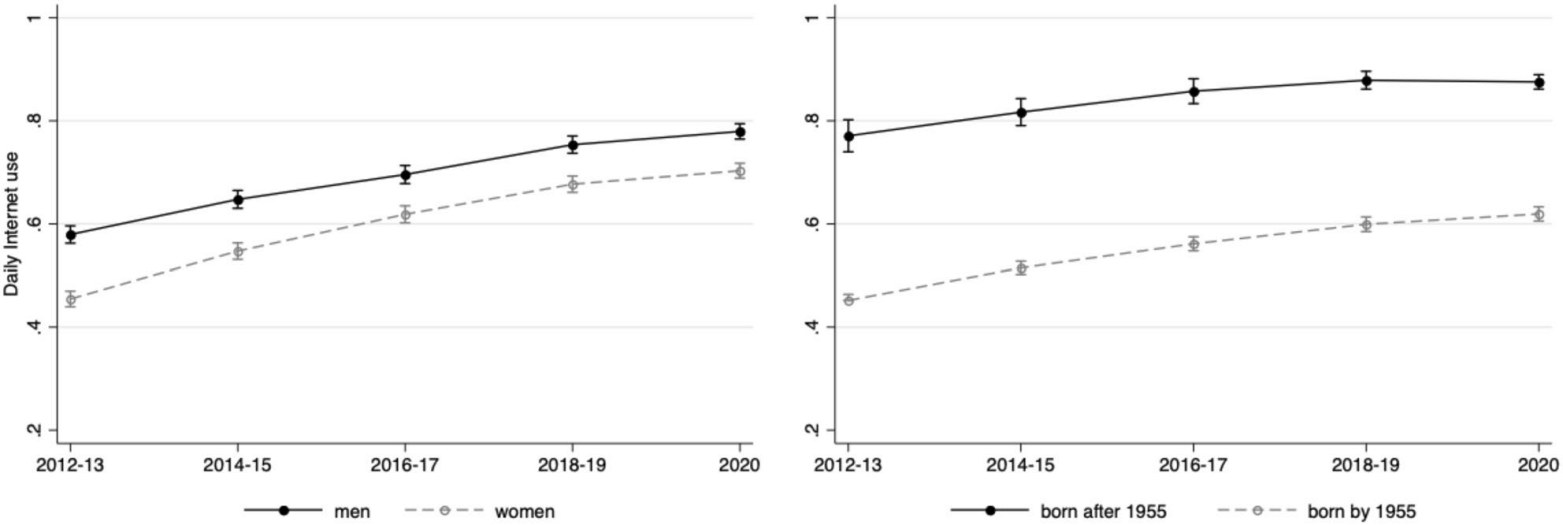
Weighted prevalence of daily Internet use over time, by gender and cohort. Estimates are from the English Longitudinal Study of Ageing, Waves 6 to 9 (2012–2019) and the COVID-19 Substudy Wave 1 (June/July 2020), weighted using cross-sectional weights at each data collection wave.

Weighted estimates from the nationally representative English Longitudinal Study of Ageing suggest that the prevalence of daily Internet use among adults aged 50 + in England increased from 52·9% in 2012/2013 (Wave 6), to 71·8% in 2018/2019 (Wave 9), and finally to 73·9% in June/July 2020 which was shortly after the outbreak of the COVID-19 pandemic (Supplementary Table S1). Figure 2 illustrates similar trends across gender and cohort, though mean levels remain substantively different throughout, where men and those under 65 were more likely to use the Internet than women and older participants, respectively. These estimates are comparable to the Office for National Statistics estimates of the prevalence of daily Internet use by age group (Supplementary Fig. S1)^47^.

When we examine within-individual changes in the likelihood of daily Internet use, we find no significant increase from 2018/2019 to June/July 2020, even when accounting for time-varying covariates, namely age, partnership and employment status, perceived financial difficulties, self-reported health, depressive symptomology, loneliness, levels of positive and negative support from partner, and contact with others. The corresponding estimate, presented in Table 2 (full estimation output in Supplementary Table S4), is a 0.2-percentage-point increase, which is not significant at the 10% level (*P*-value = 0.831). Table 2 further presents estimates from using different frequency thresholds, showing similarly no increase in likelihood of use when we alter the threshold to at least weekly or monthly use. This finding also remains unchanged when we apply an ordinal logistic model specification (after standardising the frequency variable across waves; estimated odds ratio = 1.189, *P*-value = 0.350).

**Table 2:** Within-individual changes in Internet use.

	(1) Daily Internet use		(2) At least weekly use		(3) At least monthly use	
Wave (ref: Wave 9, 2018–19)						
Wave 8 (2016–17)	− 0.007	(0.010)	− 0.008	(0.010)	− 0.002	(0.007)
COVID-19 (June-July 2020)	0.002	(0.008)	− 0.008	(0.008)	− 0.012*	(0.006)
Mean outcome at Wave 9	0.747		0.837		0.858	
Observations	13,916		13,916		13,916	
Unique individuals	5571		5571		5571	

All are binary variables unless indicated otherwise. Figures in parentheses are standard errors. *P < 0.10, **P < 0.05, ***P < 0.01.

### Factors predicting use

We further examine the pre-COVID-19 demographic, economic, and social factors predicting daily Internet use in June/July 2020, shortly after the outbreak of the pandemic. We do not look at predictors of a within-individual increase in Internet use, given that there was virtually no change. Table 1 presents the differences by daily Internet use status in June/July 2020. On average, daily users were more likely to be male, younger, not living alone, in good physical and mental health, employed, and living in less deprived neighbourhoods; and to have higher educational attainment, income, and wealth. Daily users were also less lonely and more likely to be members of an organisation.

**Figure 3:**
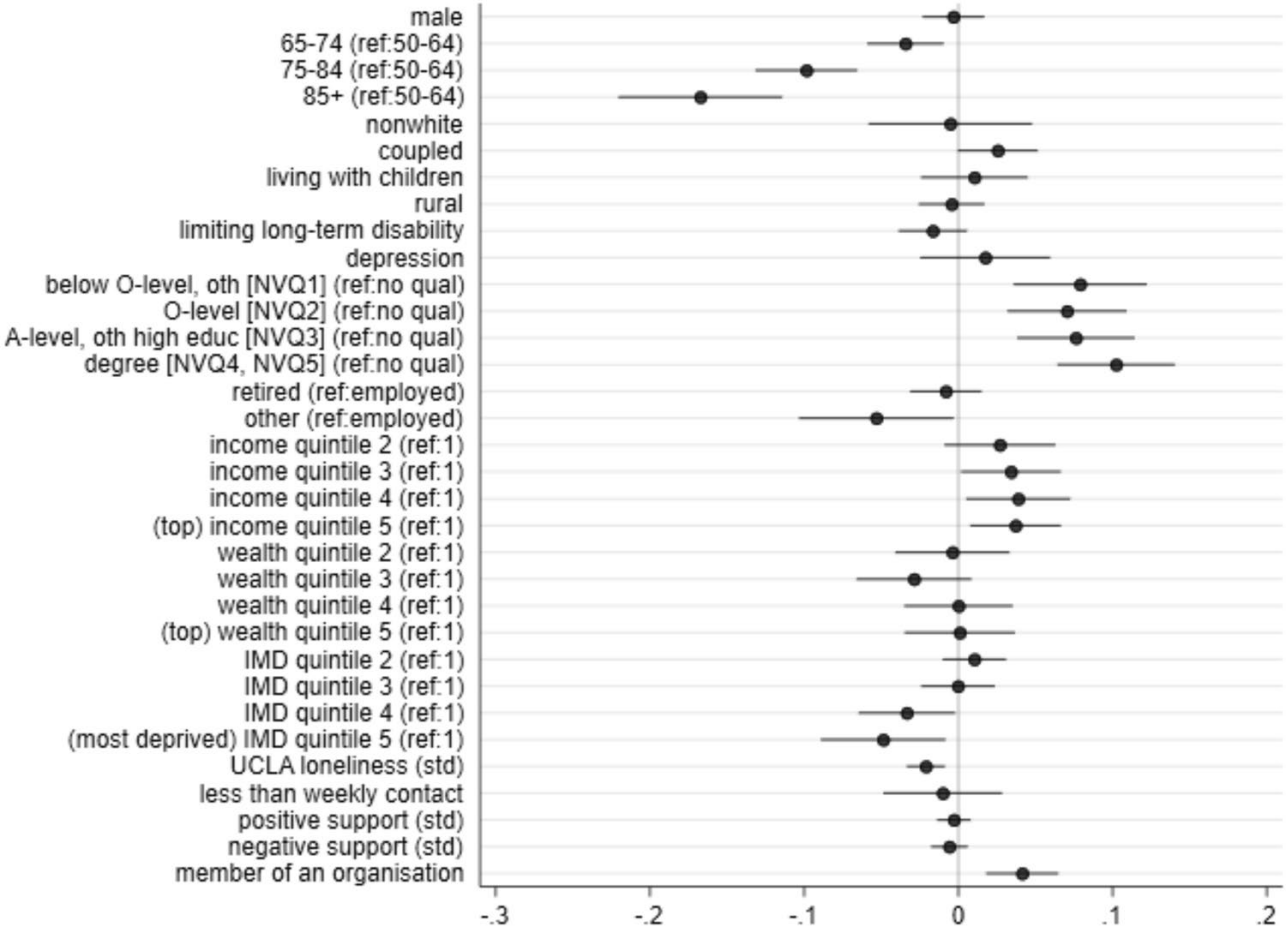
Associations between Internet use and pre-pandemic demographic, economic, and social characteristics. Estimates are from regressing daily Internet use in June/July 2020 (outcome mean 0.757) on the pre-pandemic characteristics (*n* = 5475). This regression additionally controls for pre-pandemic daily Internet use and Government Office Regions. Not shown in this figure is the estimate for past daily Internet use (coefficient estimate = 0.610, *P* < 0.01). The full regression output is in Supplementary Table S4. Std = standardised scores.

Figure 3 (full estimation output in Supplementary Table S5) illustrates a clear age gradient, even after controlling for use in 2018/2019. On average, older participants (65 +) were less likely than younger participants (50–64) to be daily Internet users shortly after the outbreak of the pandemic in June/July 2020. This discrepancy increased with age, with those aged 85 + being 17 percentage points less likely (or 22·1% relative to the June/July 2020 average) than those aged 50–64 to use the Internet daily. Partnership status was marginally associated with Internet use, where those married or in a cohabitating relationship were 3 percentage points more likely (3·4% relative to the June/July 2020 average) to be a daily Internet user. Gender, ethnicity, living with children, rurality, and health (limiting long-term disability and depressive symptomology) were not predictive of daily use.

Older adults with higher education were more likely to use the Internet daily during the pandemic in June/July 2020, with the highest prevalence of daily use observed among those with a degree (10 percentage points more than those without qualifications). Older adults who were neither employed nor retired (i.e., unemployed, sick, unoccupied) were less likely to be daily users than the employed. Those with higher income were more likely to use the Internet, where those in the top 40% of the income distribution being around 4 percentage points more likely to be daily users, compared with those in the bottom 20%. Older adults residing in the most deprived neighbourhoods were less likely, by around 5 percentage points, to be daily users, compared with the least deprived neighbourhoods. After adjusting for all these variables, wealth was not associated with the likelihood of daily Internet use.

Lonely older adults were less likely to be daily Internet users, where a one-standard deviation rise in loneliness levels was associated with a lower likelihood of daily Internet use by 2 percentage points. Older adults belonging to an organisation were more likely by 4 percentage points to be daily users than those who did not belong to an organisation. Contact with non-household members and social support were not significantly associated with daily use.

As expected, past daily Internet use (in 2018/2019) was the strongest predictor of daily Internet use during the pandemic in June/July 2020, where older adults already using the Internet daily prior to the pandemic were 61 percentage points more likely to also be daily users during the pandemic. Importantly, the demographic, economic, and social factors discussed above are estimated to be predictive (or otherwise) of daily Internet use during the pandemic in June/July 2020 after adjusting for pre-pandemic daily use, which also captures all other past observed and unobserved factors that predict Internet use more generally.

In our sensitivity analysis (Supplementary Table S5), results are qualitatively similar across different frequency thresholds of Internet use, though several patterns are worth highlighting. At lower-frequency thresholds (e.g., using the Internet at least once a week), associations between Internet use and income and neighbourhood deprivation were smaller, which could reflect the roles of these factors in providing access to better equipment and Internet connection for higher-frequency use. Associations with loneliness and organisation membership were also smaller at lower-frequency thresholds, suggesting that social connectivity via the Internet may be more apparent when used more regularly. In contrast, the association between disability status and Internet use was clearer at these lower-frequency thresholds. We also check whether these findings change when using an ordinal logistic model to account for the different frequencies of Internet use. The resulting estimates follow a very similar pattern of factors predicting Internet use, where only past use, age group, partnership status, education, household income, neighbourhood deprivation, loneliness, and organisation membership were significant predictors (Supplementary Table S6).

### Types of use

Among older adults who use the Internet at least once a month, from before to shortly after the outbreak of the pandemic, there were substantial increases in using the Internet for video or voice calls (increasing from 38·0% in 2018/2019 to 63·6% in June/July 2020), and to engage with Government services (23·7 to 41·1%). Surprisingly, Internet use to find health-related information declined markedly (80·3 to 44·5%) (Supplementary Table S2).

**Figure 4:**
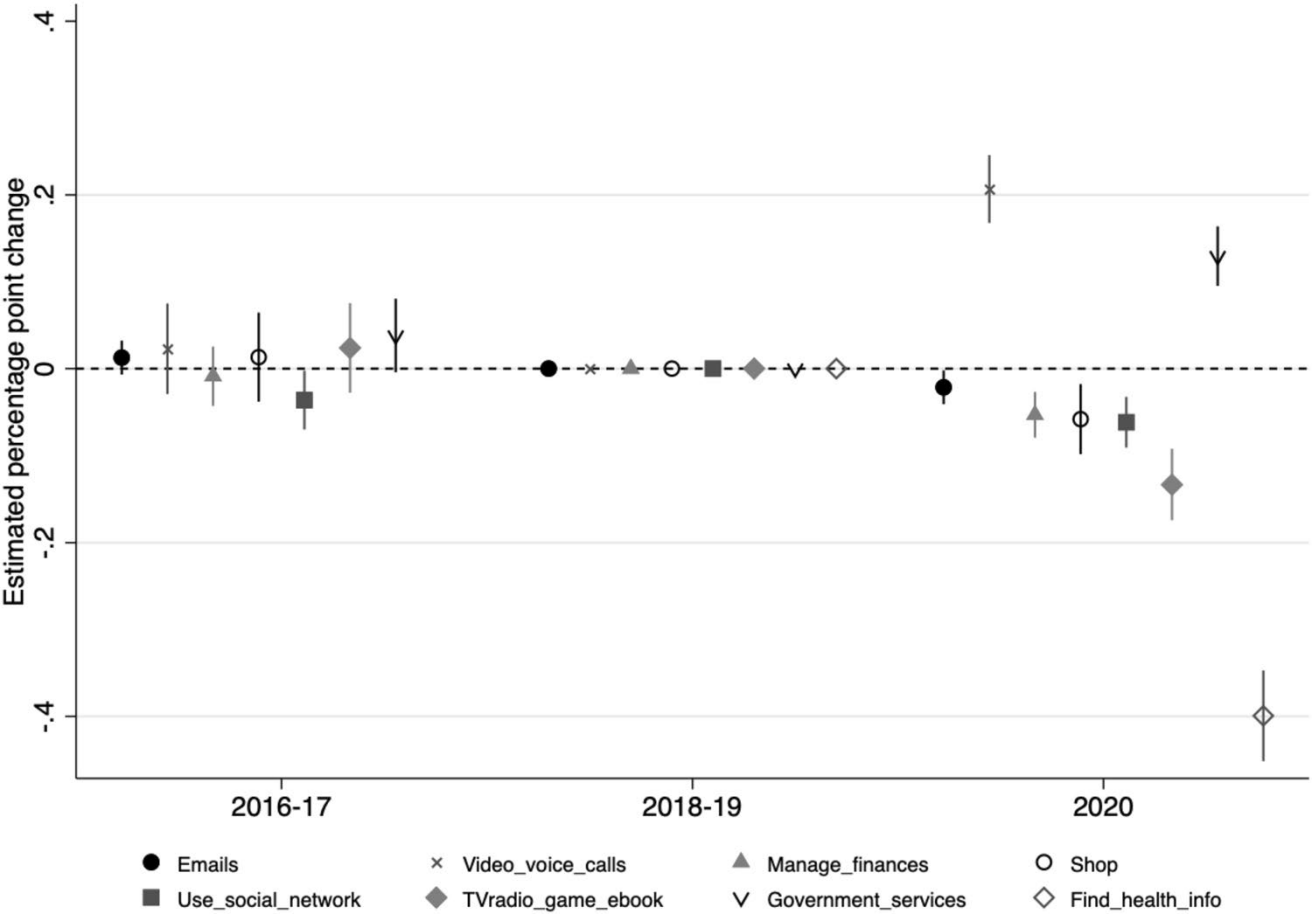
Within-individual changes from 2018/2019 in types of Internet use. Estimates are from individual fixed-effects regressions, which include controls for the following observed time-varying covariates: age, partnership and employment status, perceived financial difficulties, self-reported health, depressive symptomology, loneliness, levels of positive and negative support from partner, and contact with others. Not shown is the estimate for reading news, newspaper, or blog websites-this was not significantly different from zero, suggesting that this type of Internet use did not change during the pandemic from the 2018/19 period. The full regression output is in Supplementary Table S7 (*n* = 4831 unique individuals for types of Internet use, i.e., excluding less than monthly users).

Consistent with these descriptive findings, when examining within-individual changes and controlling for time-varying covariates (Fig. 4, full estimation output in Supplementary Table S7), there were significant increases from before (2018/2019) to shortly after the outbreak of the pandemic (June/July 2020), in using the Internet for video or voice calls (by 21 percentage points, or 55% relative to the 2018/2019 mean of 38·0%) and for information about Government services (by 13 percentage points, or 55% relative to the 2018/2019 mean of 23·7%). In contrast, Internet use for finding health-related information declined by 40 percentage points (50% relative to the 2018/2019 mean of 80·3%), and leisure use declined by 13 percentage points (21% relative to the 2018/2019 mean of 61·2%). Smaller declines were observed for other uses. We also show that the 2016/2017 estimates did not differ significantly from the reference 2018/2019 period, suggesting very little within-individual change between these two periods, with respect to the likelihood of each type of use considered.

**Figure 5:**
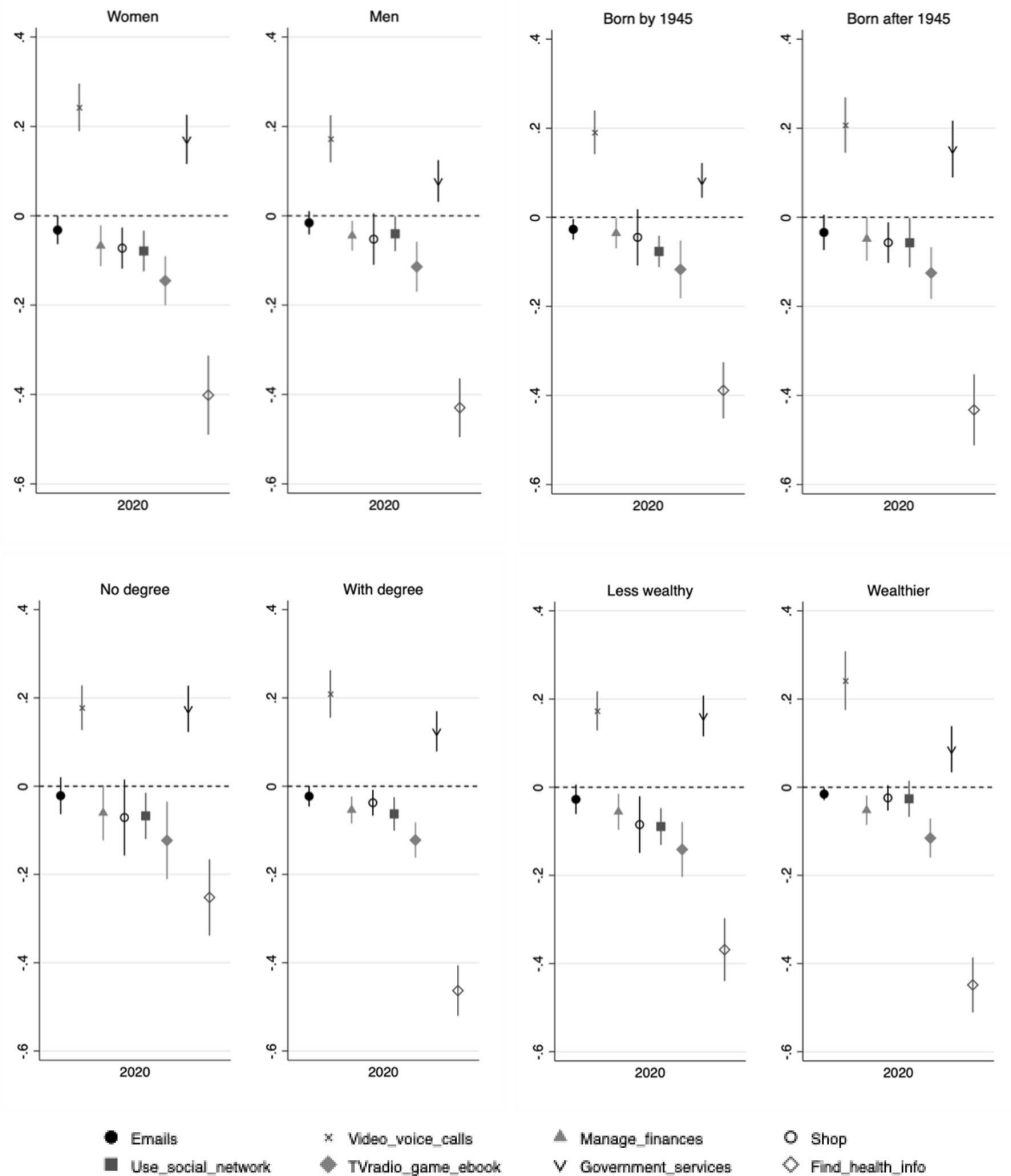
Within-individual changes from 2018/2019 in types of Internet use, by gender, cohort, education, and wealth levels. Estimates are from individual fixed-effects regressions, which include controls for the following observed time-varying covariates: age, partnership and employment status, perceived financial difficulties, self-reported health, depressive symptomology, loneliness, levels of positive and negative support from partner, and contact with others. All regression outputs are in Supplementary Table S8.

We further explore whether there was heterogeneity in these changes in types of use by gender, birth cohort, education, and wealth. Figure 5 illustrates these subgroup estimates for June/July 2020, following the specification in Fig. 4 (2016/2017 and 2018/2019 points omitted for brevity; estimation output in Supplementary Table S8). At large, across gender, birth cohort, education, and wealth subgroups, these within-individual changes were similar in direction and significance. Increased use for getting information about Government services was nevertheless more likely among women, as well as those with lower wealth, compared with their respective counterparts. Older adults with higher education (higher than A-level) were more likely than those with lower education (A-level and below) to show a decrease in Internet use for health-related information.

## Discussion

The first aim in this study is to examine changes in the frequency and types of Internet use among older adults, from before to shortly after the outbreak of the COVID-19 pandemic. Around 72% of older adults in England were already using the Internet daily in 2018/2019, rising slightly to 74% in June/July 2020 during the COVID-19 pandemic. Our longitudinal analysis reveals that on average, there were no significant within-individual changes in the likelihood of regular Internet use from 2018/2019 to June/July 2020.

To our knowledge there is little representative evidence on changes in Internet use patterns during the pandemic, although it is widely assumed that people have become more dependent on the Internet. A report from China documented a doubling in the number of Internet users aged 60 + since the beginning of the pandemic, from 6.7% to 11.2%^12^. In contrast, comparable ELSA estimates (i.e., proportion of users of any frequency, aged 60 +) suggest that in England, this remained constant at 79% before and during the pandemic. This is consistent with the general pattern of a greater digital divide in emerging economies. The lower pre-pandemic Internet use among older adults in China was also likely due to greater barriers to accessing technologies, which was addressed both formally (Government initiatives) and informally (e.g., family members helping with skills) after the outbreak^12^. We consider whether participants were unable to increase their use due to stay-at-home restrictions which may have limited their access to Internet equipment or connection. However, our conclusions remain unchanged when we control for type of device used (smartphones, computers, or both), or for places where participants accessed the Internet (home, elsewhere, or both). Compared with participants who (as reported in 2014/2015) used the Internet both at home and elsewhere (e.g., at work, education, another person’s home, on the move, library, or internet café), those who used the Internet only elsewhere were indeed less likely to have increased their likelihood of daily Internet use from before to shortly after the outbreak of the pandemic, but this was only a marginally significant difference; whereas those who used the Internet only at home were more likely to have increased their likelihood of daily use. However, neither of these three groups had a significant change in the likelihood of daily Internet use from before to shortly after the outbreak of the pandemic (Supplementary Table S9).

The second aim in this study is to document the pre-COVID-19 demographic, economic, and social factors that were predictive of Internet use shortly after the outbreak of the pandemic. That age, partnership status, education, employment, income, and neighbourhood deprivation were predictive of daily Internet use during the pandemic in June/July 2020, is consistent with other evidence on Internet use during the pandemic^14,16^, and evidence documented prior to the pandemic^4,25–37^. We also observe associations with health status at lower-frequency thresholds. In contrast with a smaller cross-sectional study in Israel (*N* = 407, conducted in April 2020)^16^, we do not find rurality to be predictive of Internet use after the outbreak of the pandemic, potentially due to the urban–rural differences in Internet access and connectivity in Israel relative to England. Unlike pre-COVID-19 studies, we also do not find gender and ethnicity to be predictive of daily Internet use during the pandemic, after accounting for other covariates and pre-COVID-19 daily use. The significant association between loneliness and Internet use provides support to discussions of their dynamic, bidirectional relationship^48^. We also find organisation membership, but not informal contact or social support, to be predictive of Internet use. This could be related to organisations moving to online platforms after the outbreak of the pandemic (e.g., live streaming of religious services and exercise classes, virtual club/association meetings), which encouraged regular Internet use among their members.

We further examine within-individual changes in types of Internet use before and shortly after the outbreak of the pandemic, which is a new contribution to existing evidence. We show that in June/July 2020, using the Internet for video or voice calls and for information about Government services increased significantly, whereas Internet use for information on health-related issues and for leisure decreased, relative to 2018/2019. These estimates of within-individual changes in daily Internet use and in the different types of uses are consistent with their corresponding general prevalence estimates. Our findings contrast with a small cross-sectional study which found an increase in Internet use during the pandemic not only for communicating but also for shopping, banking, medical appointments, reading online newspapers, using social networking services, and leisure^16^. Our representative longitudinal study adds to this evidence, confirming that there was indeed an increase in Internet-based communication after the outbreak. However, we find no increase in Internet use for shopping, reading online news, using social networking sites, managing finances, and leisure, from levels recorded prior to the outbreak (we do not observe whether they used the Internet for medical appointments).

We also observe subgroup differences, where women and lower-wealth older adults were more likely to increase Internet use for Government services-related information, compared with their counterparts. Additionally, the decline in using the Internet for health-related information was greater among older adults with higher education.

Generally, the rise in Internet use for Government services-related information is positive, given the UK Government’s effort to digitise public services^49^, which was ramped up during the pandemic^50^. This includes the development of GOV.UK and the National Health Service (NHS) Choices websites; digital strategies implemented by various local councils across the UK, such as to support the Universal Credit and Troubled Families programmes; and investments in the NHS COVID-19 app and Test and Trace programme during the pandemic. While there were concerns that older adults, especially those in financial hardship or deprived communities, would experience further exclusion^50^, that we see a general increase across all subgroups is reassuring. Older adults in lower wealth groups were in fact more likely to have increased their Internet use for information on Government services during the pandemic, compared with the pre-pandemic period, possibly for COVID-19 financial support (e.g., Coronavirus Job Retention Scheme), or to alter their pension arrangements. That older women were more likely than older men to increase their Internet use for information on Government services, is consistent with evidence that women tend to be more risk averse^51^, a personal characteristic related to perceived usefulness of information from credible sources in a time of uncertainty^52^.

The decline in Internet use to search for health-related information is surprising, considering public perception that many engaged with online information sources regarding the novel coronavirus. It may be that older adults in England received such information from friends, family, or health providers, in whom they have greater trust^53^, especially considering the intensity and pervasiveness of the COVID-19 topic in June/July 2020. Our observed increase in Internet use for communication provides some support for this explanation. An alternative explanation is that older adults may have been avoidant of COVID-19 information during this period. Data from the UK COVID-19 Social Study, which collected weekly time use data on over 75,000 adults, support this speculation^54^. At the end of March 2020, around three-quarters of older adults were spending over half an hour per day watching the news, listening to the radio or browsing the Internet for information about COVID-19, but this dropped to one-fifth by the end of July 2020. Over 10% were also tweeting, blogging, or posting content online about COVID-19 in March, declining to less than 2% by the end of July. While this could reflect a general drop in Internet use as national lockdown regulations were lifted in early July, this was not the case for Internet browsing for non-COVID-19 information, where there was only a small decline from 72% in March to 61% in July.

The positive association between education and health^55^ may explain the greater decline in Internet use for health-related information among older adults with higher education. We observe the same difference by pre-COVID-19 health, where the decline is greater among healthier older adults, compared with those with a long-term disability or depressive symptomology. This is somewhat reassuring, given that many planned treatments and medical tests were cancelled or postponed due to the pandemic (experienced by around 1 in 6 adults aged 60 + when asked in June/July 2020, from the UCL COVID-19 Social Study on over 75,000 adults, and the nationally representative UK Household Longitudinal Study on over 14,000 adults)^54,56^. The ability to self-inform on health-related issues via the Internet may have been critical during this difficult period, to manage existing health conditions in the absence of treatments and tests. In contrast with a cross-sectional US study (*N* = 5,780, conducted in mid-March 2020), which found gender and age differences in searching for and sharing information on the Internet regarding the coronavirus^14^, we do not observe these differences when considering how Internet use for health-related information changed during the pandemic.

The strength of this study is in the longitudinal nature and national representativeness of the ELSA data, and the high response rate achieved in the COVID-19 Substudy. The ability to observe the frequency and types of Internet use among older adults pre- and post-outbreak, and the ability to control for factors predicting use from the pre-outbreak period, allow us to contribute to the literature on Internet use among older adults during the pandemic, which to date consists primarily of cross-sectional studies using data collected only after the outbreak of the pandemic. Our findings should nevertheless be interpreted in light of the study's limitations: first, we rely primarily on self-reported Internet use, and there could be some differences in the understanding of ‘Internet use’ in our sample (e.g., unclear whether making voice calls from smartphones is a form of ‘Internet use’). However, as the Internet use module has also been administered in pre-COVID-19 waves, we can examine within-individual changes in use, which eliminates such time-invariant unobserved differences. Second, we are unable to examine more nuanced changes in the frequency of Internet use (e.g., from daily to more than once a day, or the change in hours spent using the Internet). It is nonetheless reassuring that our conclusions are similar across different frequency thresholds. Third, frequency and types of use may have changed over the course of the pandemic, but we only have data from one timepoint early on in the pandemic (June 3-July 26), during which the first national lockdown (March 26-July 3) was easing. It could be that there was an initial increase in Internet use earlier on in the pandemic, but as the lockdown eased, this returned to pre-COVID-19 levels. Future studies may examine longer-term changes in frequency (at a finer scale) and types of Internet use among older adults. Fourth, as with other longitudinal studies, there is non-random cumulative attrition in the ELSA sample, for which we partially correct with weights in our analysis. It is also worth noting that older adults in care homes have been excluded by design. There could be an under-representation of infrequent or non-users in our sample, in which case, the rates of Internet use would be lower than estimated.

This study documents the changes in the frequency and types of Internet use among older adults in England, from before to shortly after the outbreak of the COVID-19 pandemic, and the factors linked to use during these early days of the pandemic. Our results suggest a need to further encourage participation among older groups and those in poorer socioeconomic conditions, particularly in times of crisis. There is much to be gained from getting older adults online, including delivery of telemedicine or Internet-based interventions^57,58^. Providing training, decreasing the financial burden of Internet access and equipment, and improving usability for older adults, have been shown to be successful^59,60^; moreover, there could be a role for social ties who are regular Internet users^25^, and based on our own findings, a role for formal organisations in promoting and supporting use among their members. Such actions would be timely as we recover from the COVID-19 pandemic, a period which highlighted the importance of Internet connectivity and the provision of digital alternatives, across multiple functions of society and daily living.

## Supplementary Information

Supplementary Information.

## Data Availability

The datasets analysed are available in the UK Data Service repository as Study Numbers 5050 and 8688, https://doi.org/10.5255/UKDA-SN-5050-24; https://doi.org/10.5255/UKDA-SN-8688-3.
