# Supplementary material for: Changes in Internet use patterns among older adults in England from before to after the outbreak of the COVID-19 pandemic
Source: Sci Rep. 2023 Mar 9;13:3932. doi: 10.1038/s41598-023-30882-8 (PMC9995747; doi:10.1038/s41598-023-30882-8)
Supplement: Supplementary file 1 — Supplementary Information. [file 41598_2023_30882_MOESM1_ESM.pdf]

Changes in Internet use patterns among older adults in England from before to after the outbreak of the COVID-19 pandemic

Claryn S. J. Kung & Andrew Steptoe

Table of contents

Figure S1. Daily Internet use in the ELSA vs. Great Britain……………………………………………………..2

Table S1. Frequency of Internet use over time……………………………………………………………………3

Table S2. Types of Internet use over time……………………………………………………………………4

Table S3. Factor descriptions……………………………………………………………………………………5

Table S4. Within-individual changes in Internet use…………………………………………………………6

Table S5. Associations between Internet use and pre-COVID-19 demographic, economic, and social characteristics……………………………………………………………………………………………8

Table S6. Associations between Internet use and pre-COVID-19 characteristics: odds ratios from an ordinal logistic regression model (partial proportional odds)……………………………………………………9

Table S7. Within-individual changes in types of Internet use (Figure 4 estimates)………………………10

Table S8. Within-individual changes in types of Internet use, by gender, cohort, education, and wealth levels (Figure 5 estimates)…………………………………………………………………………………11

Table S9. Change in likelihood of daily Internet use, controlling for devices used to access the Internet and places where participants used the Internet……………………………………………………………14

Appendix references………………………………………………………………………………………15

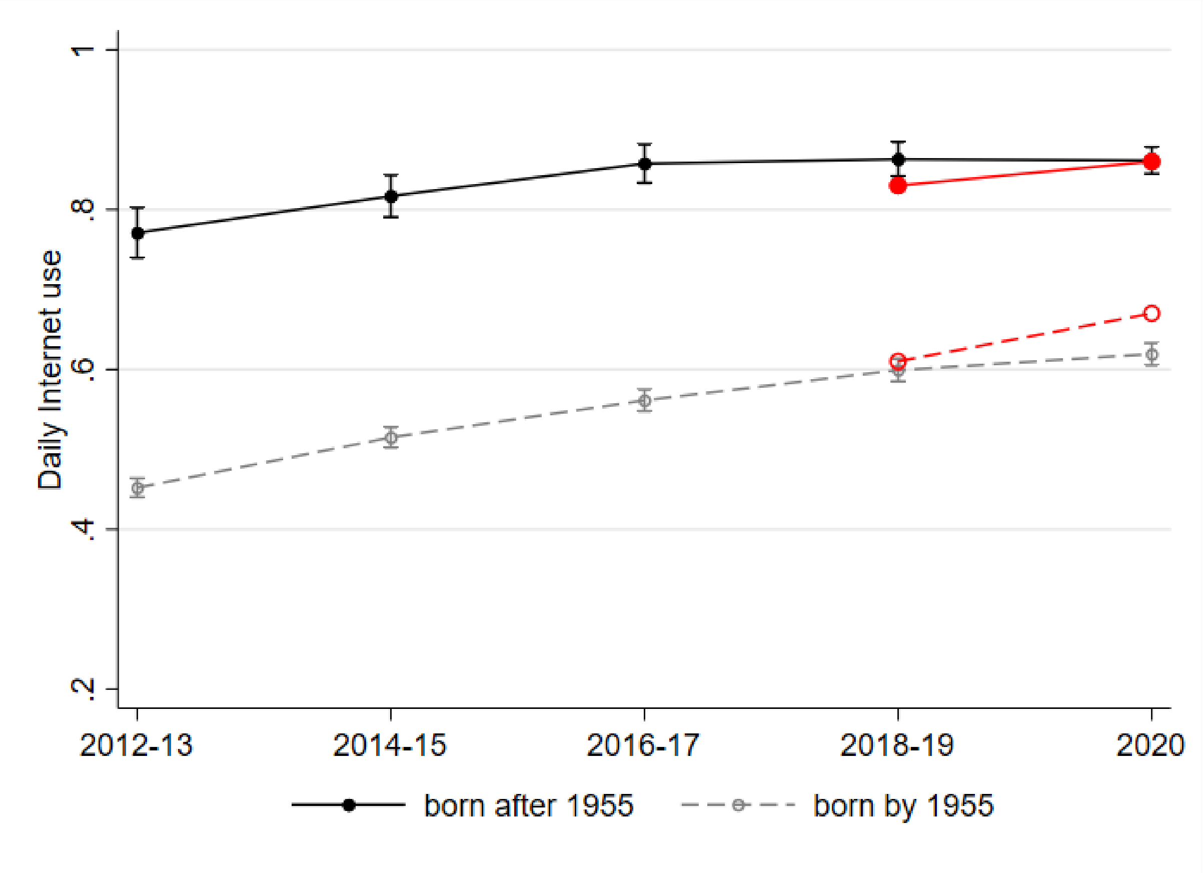

Figure S1. Daily Internet use in the ELSA vs. Great Britain.
Data from the Office for National Statistics (in red)[1] are based on the monthly Opinions and Lifestyle Survey, administered on around 1,100 participants 16 years and above in private households in Great Britain. This Figure repeats the trajectories by age plotted in Figure 1, with the inclusion of ONS estimates from 2019 and 2020, illustrating the comparability of both sets of estimates. For instance, from the ELSA, 86% of those born after 1955 and 60% of those born by 1955 used the Internet daily in 2019, whereas ONS estimates are 83% and 61%. Data pertaining to Internet access in 2020 was only collected in January and February 2020, that is, prior to the outbreak of the pandemic, giving rise to some of the minor differences seen.

Table S1. Frequency of Internet use over time.

	**Wave 6**	**Wave 7**	**Wave 8**	**Wave 9**	**COVID-19 Sub-study**
Year	2012-13	2014-15	2016-17	2018-19	June-July 2020
Frequency of use					
Every day, or almost every day	0.529	0.607	0.662	0.718	
At least once a week (but not every day)	0.122	0.112	0.097	0.092	
At least once a month (but not every week)	0.035	0.032	0.026	0.022	
At least once every 3 months	0.011	0.009	0.007	0.007	
Less than every 3 months	0.016	0.014	0.016	0.011	
Never	0.287	0.226	0.191	0.149	
More than once a day					0.517
Every day, or almost every day					0.222
At least once a week (but not every day)					0.074
At least once a month (but not every week)					0.018
Less than monthly					0.017
Never					0.152
N	7,752	6,909	6,123	6,202	6,840

Notes: Figures are averages, weighted using cross-sectional weights provided by the ELSA team. In ELSA Waves 1 through 5, participants were asked whether the statement “I use the Internet and/or email” applied to them (binary variable without frequency options).

Table S2. Types of Internet use over time.

	Wave 8	Wave 9	COVID-19 Sub-study
Year	2016-17	2018-19	June-July 2020
Types of use			
Sending/receiving e-mails	0.891	0.899	0.881
Voice or video calls	0.316	0.380	0.636
Finding information on health-related issues		0.803	0.445
Searching for information for learning, research, fact finding [a]	0.901	0.605	
Finances (banking, paying bills)	0.604	0.651	0.615
Shopping/buying goods or services	0.758	0.776	0.735
Selling goods or services [a]	0.111	0.131	
Using social networking sites	0.485	0.559	0.518
Creating, uploading, or sharing content [a]	0.114	0.106	
News/newspaper/blog websites	0.512	0.562	0.576
Streaming/downloading TV/radio, music, games, or ebooks [b]	0.578	0.612	0.515
Looking for a job or sending a job application [a]	0.103	0.099	
Getting information about Government services (obtaining benefits, paying taxes, driving licence or passport, etc) [c]	0.232	0.237	0.411
Other [a]	0.074	0.076	
None of the above	0.004	0.003	0.007
N	4,726	5,026	5,685

Notes: Figures are averages, weighted using cross-sectional weights provided by the ELSA team. Participants are only included if they have used the Internet at least once a month, to be consistent with the coding in the COVID-19 Substudy.

Due to differences in the options presented over the different ELSA waves, we collapse or remove several categories from earlier ELSA Waves to make them consistent with the options presented in the COVID-19 Substudy:

[a] Not asked in the COVID-19 Substudy, so we do not further analyse this.
[b] In Waves 8 and 9, this was presented as two separate items, namely "streaming/downloading live or on demand TV/radio, music (iTunes, Spotify), or ebooks" and "games". We collapse these two items to be consistent with the COVID-19 Substudy version.
[c] In Waves 8 and 9, this was presented as "using public services" cf. "getting information about Government services".

Table S3. Factor descriptions.

Factor	Description
Has depressive symptomatology	Depressive symptomology is measured using the eight-item Center for Epidemiologic Studies Depression Scale (CES-D), where participants are asked whether, "much of the time during the past week", they felt depressed, felt that everything they did was an effort, their sleep was restless, they were happy, they felt lonely, they enjoyed life, they felt sad, and they could not get going. We code every positive response as 1 (reverse-coded for "felt happy" and "enjoyed life"), so the summary CES-D score takes on a range between 0 and 8. Following other studies we use a score of 4 or greater to indicate marked symptomology.[2]
UCLA Loneliness score	The UCLA Loneliness score is based on the three items "How often do you feel you lack companionship?", "How often do you feel left out?", and "How often do you feel isolated from others?", where participants can respond "hardly ever or never", "some of the time", or "often". We sum the responses to obtain a loneliness score ranging from 3 to 9, where higher scores represent greater loneliness. Before this score is included in the regression analysis, it is first standardised to have mean 0 and standard deviation 1, such that the estimated coefficient would represent changes in the outcome variable when the score changes by 1 standard deviation.
Positive / negative support	Positive support is captured by the items "How much do they really understand the way you feel about things?", "How much can you rely on them if you have a serious problem?", and "How much can you open up to them if you need to talk about your worries?", whereas negative support is captured by the items "How much do they criticise you?", "How much do they let you down when you are counting on them?", "How much do they get on your nerves?", and "How often do they make too many demands on you?". These seven support questions, where response options are "a lot", "some, "a little", and "not at all", are presented separately for partners, children, other immediate family, and friends. We reverse-code each item, take the average positive support ratings (across three items), and average this score across all four relationships. The same is done for negative support (across four items, and across all four relationships). Our final positive and negative support scores therefore range from 0 to 3, with higher scores representing higher levels of the respective type of support. Before these two scores are included in the regression analysis, the scores are first standardised to have mean 0 and standard deviation 1, such that their estimated coefficients would represent changes in the outcome variable when the scores change by 1 standard deviation.
At least weekly contact with non-household members	"Contact with non-household members" reflects meet up (including both arranged and chance meetings), speak on the phone, write or email, or send or receive text messages; with children, other immediate family, or friends who are not living with them.
Member of an organisation	Examples of organisations, clubs, or societies participants may be members of are political parties, tenant/resident groups, church, gyms, etc.

Table S4. Within-individual changes in Internet use.

	(1) Daily Internet use		(2) At least weekly use		(3) At least monthly use	
Wave (ref: Wave 9, 2018-19)						
Wave 8 (2016-17)	-0.007	(0.010)	-0.008	(0.010)	-0.002	(0.007)
COVID-19 (June-July 2020)	0.002	(0.008)	-0.008	(0.008)	-0.012*	(0.006)
Age	0.002	(0.003)	0.002	(0.004)	0.002	(0.003)
Married/cohabitating	-0.040	(0.031)	-0.013	(0.021)	-0.035	(0.022)
Employment (ref: employed)						
Retired	0.009	(0.015)	-0.006	(0.010)	-0.001	(0.009)
Other	-0.000	(0.017)	0.001	(0.011)	-0.001	(0.009)
Financial difficulties [a]	0.029	(0.018)	-0.008	(0.012)	0.010	(0.010)
Self-rated health [b]	0.017	(0.011)	-0.007	(0.010)	-0.000	(0.010)
Has depressive symptomology [c]	0.008	(0.012)	-0.008	(0.009)	-0.004	(0.008)
UCLA Loneliness score [d]	0.001	(0.006)	0.004	(0.005)	0.007	(0.005)
Positive support score [d]	-0.006	(0.013)	0.001	(0.009)	0.002	(0.010)
Negative support score [d]	0.011*	(0.006)	0.000	(0.005)	0.002	(0.005)
Less than weekly contact with non-household members [e]	-0.013	(0.023)	-0.013	(0.017)	-0.014	(0.019)
Mean outcome at Wave 9	0.747		0.837		0.858	
Observations	13916		13916		13916	
Unique individuals	5571		5571		5571	

Notes: All explanatory variables are binary variables unless indicated otherwise. $*p<0.10$, $**p<0.05$, $***p<0.01$. Figures in parentheses are standard errors.

[a] We do not observe participants' income values in the COVID-19 Substudy, so we use perceived financial difficulties. For Waves 8 and 9, this is based on the question "Which best describes how you and your partner are getting along financially these days?", where possible responses are "manage very well", "manage quite well", "get by alright", "don't manage very well", "have some financial difficulties", and "have severe financial difficulties". Perceived financial difficulties is defined as selecting one of the latter three options. In the COVID-19 Substudy, two questions are used: (A) "In the 3 months before the coronavirus outbreak, how well were you managing financially?", to which possible responses are (1) "living comfortably", (2) "doing all right", (3)"just about getting by", (4) "finding it quite difficult", and (5) "finding it very difficult"; and (B) "How do you feel your current financial situation compares to before the coronavirus outbreak?", to which possible responses are (1) "I'm much worse off", (2) "I'm a little worse off", (3) "I'm about the same", (4) "I'm a little better off", and (5) "I'm much better off". Perceived financial difficulties indicates combinations (A4 or A5) and (B1, B2, or B3), (A2 or A3) and (B1 or B3), or (A1) and (B1).

[b] We do not observe whether participants have a limiting, long-standing illness in the COVID-19 Substudy, so we use self-reported health, where participants are asked whether they would say their health is "excellent", "very good", "good", "fair", or "poor". We dichotomise this variable to indicate good health (that is, selecting the first three response options).

[c] For consistency with the Center for Epidemiologic Studies Depression Scale items administered in the COVID-19 Substudy, only seven items are considered across all three waves. Participants are asked whether, "much of the time during the past week", they felt depressed, felt that everything they did was an effort, their sleep was restless, they were happy, they felt lonely, they enjoyed life, and they could not get going (i.e., excluding "felt sad"). We code every positive response as 1 (reverse-coded for "felt happy" and "enjoyed life"), so the summary CES-D score takes on a range between 0 and 7. We use a score of 3 or greater to indicate marked depressive symptomology.[3]

[d] The UCLA Loneliness score is based on the three items "How often do you feel you lack companionship?", "How often do you feel left out?", and "How often do you feel isolated from others?", where participants can respond "hardly ever or never", "some of the time", or "often". We sum the responses to obtain a loneliness score ranging from 3 to 9, where higher scores represent greater loneliness. Positive support is captured by the items "How much do they really understand the way you feel about things?", "How much can you rely on them if you have a serious problem?", and "How much can you open up to them if you need to talk about your worries?", whereas negative support is captured by the items "How much do they criticise you?", "How much do they let you down when you are counting on them?", "How much do they get on your nerves?", and "How often do they make too many demands on you?". These seven support questions, where response options are "a lot", "some, "a little", and "not at all", are presented only for partners in the COVID-19 Substudy, so for consistency we consider only positive and negative support for partners across all three waves. We reverse-code each item, take the average positive support ratings (across three items), and the same is done for negative support (across four items). Our final positive and negative support scores therefore range from 0 to 3, with higher scores representing higher

levels of the respective type of support. Before these three scores are included in the regression analysis, the scores are first standardised to have mean 0 and standard deviation 1, such that their estimated coefficients would represent changes in the outcome variable when the scores change by 1 standard deviation.

[e] In the COVID-19 Substudy, “contact with non-household members” reflects speak on the phone, video-calling, write or email, or send or receive text messages; with children, other immediate family, or other relatives or friends who are not living with them (i.e., participants are not asked regarding “meet up”, as in previous waves).

Table S5. Associations between Internet use and pre-COVID-19 demographic, economic, and social characteristics.

	**(1) Daily use**		**(2) At least weekly use**		**(3) At least monthly use**	
Past Internet use (corresponding threshold)	0.610***	(0.018)	0.715***	(0.018)	0.737***	(0.019)
Demographic						
Male	-0.003	(0.010)	-0.012	(0.008)	-0.008	(0.007)
Age group (ref: 50-64 years)						
65-74 years	-0.034***	(0.013)	-0.034***	(0.010)	-0.031***	(0.010)
75-84 years	-0.099***	(0.017)	-0.058***	(0.014)	-0.060***	(0.013)
85+ years (ref: 50-64)	-0.167***	(0.027)	-0.110***	(0.026)	-0.091***	(0.025)
Non-white	-0.005	(0.027)	0.031	(0.020)	0.028	(0.017)
Married/cohabitating	0.026*	(0.013)	0.032***	(0.010)	0.026***	(0.009)
Living with children	0.010	(0.018)	-0.008	(0.015)	-0.014	(0.013)
Government Office Region (ref: South East)						
North East	0.000	(0.023)	0.010	(0.020)	0.001	(0.021)
North West	-0.029	(0.019)	-0.016	(0.016)	0.006	(0.014)
Yorkshire and The Humber	-0.010	(0.021)	-0.003	(0.013)	-0.012	(0.012)
East Midlands	-0.031*	(0.018)	-0.029**	(0.012)	-0.029**	(0.012)
West Midlands	-0.020	(0.021)	-0.020	(0.015)	-0.025*	(0.014)
East of England	-0.006	(0.017)	-0.021*	(0.012)	-0.017*	(0.010)
London	-0.024	(0.021)	-0.021	(0.016)	-0.001	(0.015)
South West	-0.007	(0.017)	0.003	(0.012)	0.005	(0.011)
Live in rural area	-0.004	(0.011)	-0.002	(0.009)	0.001	(0.008)
Has a limiting, long-term disability	-0.017	(0.011)	-0.020**	(0.009)	-0.018**	(0.009)
Has depressive symptomology	0.017	(0.022)	0.020	(0.015)	0.005	(0.013)
Economic						
Education (ref: no qualifications)						
Below O-level, foreign/other	0.079***	(0.022)	0.020	(0.020)	0.020	(0.019)
O-level	0.070***	(0.020)	0.033*	(0.017)	0.042**	(0.016)
A-level, other higher degree (NVQ3)	0.076***	(0.019)	0.062***	(0.017)	0.063***	(0.016)
Degree (NVQ4, NVQ5)	0.102***	(0.019)	0.065***	(0.016)	0.060***	(0.016)
Employment status (ref: employed)						
Retired	-0.008	(0.012)	0.001	(0.010)	-0.003	(0.010)
Other (unemployed, sick, unoccupied)	-0.053**	(0.026)	-0.009	(0.019)	0.017	(0.020)
Household income quintile (ref: 1)						
2	0.027	(0.018)	0.005	(0.014)	0.003	(0.014)
3	0.034**	(0.016)	0.007	(0.013)	0.013	(0.014)
4	0.039**	(0.017)	0.007	(0.012)	0.021*	(0.011)
5 (top)	0.037**	(0.015)	0.007	(0.012)	0.018*	(0.011)
Wealth quintile (ref: 1)						
2	-0.004	(0.019)	0.013	(0.013)	0.019	(0.013)
3	-0.029	(0.019)	-0.003	(0.013)	0.011	(0.012)
4	0.000	(0.018)	-0.001	(0.014)	-0.001	(0.013)
5 (top)	0.001	(0.018)	0.004	(0.015)	0.011	(0.013)
Index of Multiple Deprivation quintile (ref: 1, least deprived)						
2	0.010	(0.011)	-0.005	(0.008)	-0.002	(0.007)
3	-0.000	(0.012)	-0.012	(0.010)	-0.003	(0.008)
4	-0.033**	(0.016)	-0.025**	(0.012)	-0.026**	(0.012)
5 (most deprived)	-0.049**	(0.021)	-0.043**	(0.017)	-0.016	(0.016)
Social						
UCLA Loneliness score [a]	-0.021***	(0.006)	-0.011**	(0.005)	-0.008*	(0.004)
Less than weekly contact with non-household members	-0.010	(0.020)	0.003	(0.035)	-0.018	(0.036)
Positive support score [a]	-0.003	(0.006)	-0.004	(0.004)	-0.001	(0.004)
Negative support score [a]	-0.006	(0.006)	-0.008	(0.005)	-0.002	(0.005)
Member of an organisation	0.041***	(0.012)	0.034***	(0.009)	0.016*	(0.009)
Mean outcome (likelihood of daily Internet use)	0.757		0.830		0.848	
N (weighted)	5,475		5,475		5,475	
R^2	0.558		0.631		0.653	

Notes: All are binary variables unless indicated otherwise. All income and wealth variables are recorded at the benefit unit level. *p<0.10, **p<0.05, ***p<0.01. Figures in parentheses are standard errors.
[a] Score standardised to mean 0 and standard deviation 1.

Table S6. Associations between Internet use and pre-COVID-19 characteristics: odds ratios from an ordinal logistic regression model (partial proportional odds).

Daily user: more than once a day/ every day<br>Irregular user: at least once a week/ at least once a month<br>Non-user: less than monthly/ never	**(1) Daily vs. irregular or non-user**		**(2) Daily or irregular vs. non-user**	
Outcome at Wave 9 (ref: non-user)				
Irregular user	7.209***	(1.391)	19.619***	(3.403)
Daily user	104.196***	(19.127)	184.693***	(40.301)
Demographic				
Male	0.999	(0.129)	0.744*	(0.114)
Age group (ref: 50-64 years)				
65-74 years	0.492***	(0.085)	0.492***	(0.085)
75-84 years	0.328***	(0.064)	0.328***	(0.064)
85+ years (ref: 50-64)	0.258***	(0.071)	0.258***	(0.071)
Non-white	0.995	(0.324)	0.995	(0.324)
Married/cohabitating	1.458***	(0.192)	1.458***	(0.192)
Living with children	1.149	(0.459)	1.149	(0.459)
Government Office Region (ref: South East)				
North East	1.126	(0.310)	1.126	(0.310)
North West	0.742	(0.167)	1.390	(0.442)
Yorkshire and The Humber	0.821	(0.187)	0.821	(0.187)
East Midlands	0.663*	(0.140)	0.663*	(0.140)
West Midlands	0.798	(0.181)	0.798	(0.181)
East of England	0.875	(0.165)	0.875	(0.165)
London	0.858	(0.232)	0.858	(0.232)
South West	0.998	(0.218)	0.998	(0.218)
Live in rural area	0.961	(0.131)	0.961	(0.131)
Has a limiting, long-term disability	0.836	(0.100)	0.836	(0.100)
Has depressive symptomology	1.078	(0.217)	1.078	(0.217)
Economic				
Education (ref: no qualifications)				
Below O-level, foreign/other	1.410*	(0.282)	1.410*	(0.282)
O-level	1.277	(0.230)	1.277	(0.230)
A-level, other higher degree (NVQ3)	1.353*	(0.246)	2.486***	(0.526)
Degree (NVQ4, NVQ5)	2.516***	(0.527)	2.516***	(0.527)
Employment status (ref: employed)				
Retired	1.110	(0.176)	0.660*	(0.144)
Other (unemployed, sick, unoccupied)	0.727	(0.186)	0.727	(0.186)
Household income quintile (ref: 1)				
2	1.171	(0.205)	1.171	(0.205)
3	1.209	(0.205)	1.209	(0.205)
4	1.275	(0.229)	1.275	(0.229)
5 (top)	1.728***	(0.305)	1.728***	(0.305)
Wealth quintile (ref: 1)				
2	1.119	(0.207)	1.119	(0.207)
3	0.780	(0.153)	1.194	(0.273)
4	0.996	(0.190)	0.996	(0.190)
5 (top)	1.102	(0.230)	1.102	(0.230)
Index of Multiple Deprivation quintile (ref: 1, least deprived)				
2	1.128	(0.160)	1.128	(0.160)
3	0.983	(0.157)	0.983	(0.157)
4	0.639**	(0.114)	0.639**	(0.114)
5 (most deprived)	0.640**	(0.135)	0.640**	(0.135)
Social				
UCLA Loneliness score [a]	0.827***	(0.053)	0.827***	(0.053)
Less than weekly contact with non-household members	1.098	(0.408)	1.098	(0.408)
Positive support score [a]	1.019	(0.063)	1.019	(0.063)
Negative support score [a]	0.950	(0.065)	0.950	(0.065)
Member of an organisation	1.451***	(0.183)	1.451***	(0.183)

Notes: All are binary variables unless indicated otherwise. All income and wealth variables are recorded at the benefit unit level. *p<0.10, **p<0.05, ***p<0.01. Figures in parentheses are standard errors. For each variable, the decision criterion for whether the proportional odds assumption is imposed is set to alpha=0.05. The resulting model, where the constraint is relaxed for past use frequency, gender, Government Office Region, education level, employment status, and wealth quintile, does not violate the proportional odds assumption. Weighted N=5,475, of which proportions of daily, weekly or monthly, and less than monthly or never use, are 0.757, 0.092, and 0.156.
[a] Score standardised to mean 0 and standard deviation 1.

Table S7. Within-individual changes in types of Internet use (Figure 4 estimates).

	**(1) Daily Internet Use**		**(2) Sending/ receiving emails**		**(3) Voice or video calls**		**(4) Managing finances**		**(5) Shopping/buying goods or services**	
Wave (ref: Wave 9, 2018-19)										
Wave 8 (2016-17)	-0.007	(0.010)	0.013	(0.010)	0.023	(0.027)	-0.009	(0.017)	0.013	(0.026)
COVID-19 (June-July 2020)	0.002	(0.008)	-0.022**	(0.010)	0.207***	(0.020)	-0.053***	(0.013)	-0.058***	(0.021)
Age	0.002	(0.003)	0.004	(0.005)	0.031***	(0.011)	0.007	(0.007)	0.008	(0.012)
Married/cohabitating	-0.040	(0.031)	-0.011	(0.031)	-0.017	(0.060)	-0.047	(0.038)	0.044	(0.049)
Employment (ref: employed)										
Retired	0.009	(0.015)	0.006	(0.012)	0.018	(0.025)	0.010	(0.019)	0.013	(0.018)
Other	-0.000	(0.017)	0.002	(0.016)	0.048*	(0.029)	-0.006	(0.022)	0.008	(0.022)
Financial difficulties [a]	0.029	(0.018)	0.019	(0.018)	-0.053**	(0.026)	-0.000	(0.028)	-0.042	(0.026)
Self-rated health [b]	0.017	(0.011)	-0.005	(0.013)	0.004	(0.022)	-0.024	(0.016)	0.023	(0.017)
Has depressive symptomology [c]	0.008	(0.012)	-0.009	(0.012)	0.014	(0.021)	0.007	(0.019)	-0.008	(0.018)
UCLA Loneliness score [d]	0.001	(0.006)	-0.006	(0.005)	0.013	(0.010)	-0.004	(0.007)	0.011	(0.008)
Positive support score [d]	-0.006	(0.013)	-0.006	(0.012)	-0.015	(0.025)	-0.014	(0.015)	-0.015	(0.019)
Negative support score [d]	0.011*	(0.006)	-0.001	(0.007)	-0.007	(0.012)	0.013	(0.008)	0.012	(0.009)
Less than weekly contact with non-household members [e]	-0.013	(0.023)	-0.007	(0.029)	-0.187***	(0.041)	0.040	(0.034)	0.053	(0.035)
Mean outcome at Wave 9	0.747		0.901		0.379		0.653		0.782	
Observations	13916		11610		11610		11610		11610	
Unique individuals	5571		4831		4831		4831		4831	

	**(6) Using social networking sites**		**(7) Reading news/ newspaper/ blog websites**		**(8) Streaming/ downloading TV/ radio, music, games, or ebooks**		**(9) Getting information about Government services**		**(10) Finding information on health-related issues**	
Wave (ref: Wave 9, 2018-19)										
Wave 8 (2016-17)	-0.036**	(0.017)	-0.014	(0.020)	0.024	(0.026)	0.038*	(0.022)		
COVID-19 (June-July 2020)	-0.062***	(0.015)	-0.007	(0.015)	-0.133***	(0.021)	0.130***	(0.017)	-0.399***	(0.027)
Age	0.006	(0.007)	0.002	(0.008)	0.011	(0.011)	0.013	(0.009)	0.013	(0.015)
Married/cohabitating	0.080*	(0.048)	0.024	(0.062)	0.065	(0.063)	-0.002	(0.071)	0.017	(0.080)
Employment (ref: employed)										
Retired	0.021	(0.018)	-0.007	(0.023)	0.028	(0.023)	0.033	(0.029)	-0.011	(0.038)
Other	0.046**	(0.020)	-0.008	(0.027)	0.024	(0.028)	0.047	(0.031)	0.008	(0.039)
Financial difficulties [a]	-0.014	(0.025)	0.031	(0.032)	0.013	(0.030)	0.056*	(0.033)	0.010	(0.043)
Self-rated health [b]	0.008	(0.019)	-0.028	(0.023)	-0.041**	(0.021)	0.005	(0.022)	-0.065**	(0.032)
Has depressive symptomology [c]	-0.008	(0.018)	-0.018	(0.020)	0.002	(0.020)	0.072***	(0.023)	0.018	(0.028)
UCLA Loneliness score [d]	0.014*	(0.008)	-0.000	(0.011)	-0.004	(0.010)	0.003	(0.011)	0.003	(0.013)
Positive support score [d]	-0.033	(0.021)	-0.004	(0.023)	-0.015	(0.027)	0.004	(0.028)	-0.011	(0.035)
Negative support score [d]	0.004	(0.010)	0.015	(0.011)	-0.014	(0.012)	0.010	(0.012)	0.010	(0.016)
Less than weekly contact with non-household members [e]	-0.089**	(0.036)	0.053	(0.037)	-0.048	(0.052)	-0.080	(0.060)	-0.025	(0.059)
Mean outcome at Wave 9	0.563		0.565		0.619		0.242		0.803	
Observations	11610		11610		11610		11610		8624	
Unique individuals	4831		4831		4831		4831		4766	

Notes: All are binary variables unless indicated otherwise. *p<0.10, **p<0.05, ***p<0.01. Figures in parentheses are standard errors. See Table S4 for notes a-e.

Table S8. Within-individual changes in types of Internet use, by gender, cohort, education, and wealth levels (Figure 5 estimates).

	**(1) Daily Internet Use**		**(2) Sending/ receiving emails**		**(3) Voice or video calls**		**(4) Managing finances**		**(5) Shopping/buying goods or services**	
Women (ref: Wave 9)										
Wave 8	-0.006	(0.012)	0.022	(0.022)	0.004	(0.038)	0.002	(0.031)	0.032	(0.031)
COVID-19	0.012	(0.009)	-0.032**	(0.016)	0.242***	(0.027)	-0.067***	(0.023)	-0.072***	(0.023)
Time varying covariates	Yes		Yes		Yes		Yes		Yes	
Mean outcome at Wave 9	0.711		0.896		0.390		0.625		0.769	
Observations	7374		5970		5970		5970		5970	
Unique individuals	2945		2484		2484		2484		2484	
Men (ref: Wave 9)										
Wave 8	-0.012	(0.016)	0.007	(0.009)	0.036	(0.034)	-0.017	(0.022)	0.005	(0.036)
COVID-19	-0.007	(0.013)	-0.016	(0.013)	0.172***	(0.027)	-0.045***	(0.017)	-0.052*	(0.029)
Time varying covariates	Yes		Yes		Yes		Yes		Yes	
Mean outcome at Wave 9	0.787		0.907		0.368		0.682		0.796	
Observations	6542		5639		5639		5639		5639	
Unique individuals	2626		2346		2346		2346		2346	
Born by 1945 (ref: Wave 9)										
Wave 8	-0.004	(0.013)	0.004	(0.012)	0.054	(0.033)	0.023	(0.021)	0.045	(0.039)
COVID-19	0.019*	(0.011)	-0.027**	(0.012)	0.191***	(0.025)	-0.036**	(0.017)	-0.045	(0.032)
Time varying covariates	Yes		Yes		Yes		Yes		Yes	
Mean outcome at Wave 9	0.473		0.859		0.224		0.451		0.620	
Observations	3534		2139		2139		2139		2139	
Unique individuals	1340		865		865		865		865	
Born after 1945 (ref: Wave 9)										
Wave 8	-0.016	(0.023)	0.033	(0.023)	0.017	(0.043)	-0.027	(0.034)	-0.001	(0.030)
COVID-19	0.002	(0.017)	-0.034*	(0.020)	0.207***	(0.032)	-0.048*	(0.025)	-0.057**	(0.023)
Time varying covariates	Yes		Yes		Yes		Yes		Yes	
Mean outcome at Wave 9	0.831		0.910		0.412		0.695		0.816	
Observations	10382		9471		9471		9471		9471	
Unique individuals	4230		3965		3965		3965		3965	
No degree (ref: Wave 9)										
Wave 8	-0.034*	(0.018)	0.033	(0.024)	-0.019	(0.039)	-0.027	(0.036)	0.097*	(0.059)
COVID-19	0.008	(0.016)	-0.022	(0.021)	0.178***	(0.026)	-0.060*	(0.032)	-0.071	(0.044)
Time varying covariates	Yes		Yes		Yes		Yes		Yes	
Mean outcome at Wave 9	0.525		0.752		0.299		0.475		0.613	
Observations	4042		2590		2590		2590		2590	
Unique individuals	1635		1143		1143		1143		1143	
With degree (ref: Wave 9)										
Wave 8	0.014	(0.013)	0.008	(0.012)	0.043	(0.036)	0.002	(0.020)	-0.028	(0.018)
COVID-19	-0.007	(0.010)	-0.023*	(0.012)	0.209***	(0.027)	-0.054***	(0.016)	-0.037**	(0.015)

Time varying covariates	Yes		Yes		Yes		Yes		Yes	
Mean outcome at Wave 9	0.830		0.943		0.402		0.703		0.830	
Observations	9872		9017		9017		9017		9017	
Unique individuals	3935		3687		3687		3687		3687	
Less wealthy (ref: Wave 9)										
Wave 8	-0.012	(0.014)	0.020	(0.016)	-0.002	(0.029)	-0.017	(0.027)	0.036	(0.042)
COVID-19	-0.005	(0.012)	-0.028	(0.017)	0.173***	(0.023)	-0.056***	(0.021)	-0.085***	(0.033)
Time varying covariates	Yes		Yes		Yes		Yes		Yes	
Mean outcome at Wave 9	0.684		0.857		0.338		0.606		0.731	
Observations	8021		6222		6222		6222		6222	
Unique individuals	3284		2687		2687		2687		2687	
Wealthier (ref: Wave 9)										
Wave 8	0.003	(0.014)	0.004	(0.010)	0.061	(0.046)	0.007	(0.021)	-0.013	(0.019)
COVID-19	0.012	(0.011)	-0.016**	(0.007)	0.242***	(0.034)	-0.052***	(0.017)	-0.024*	(0.015)
Time varying covariates	Yes		Yes		Yes		Yes		Yes	
Mean outcome at Wave 9	0.831		0.955		0.427		0.707		0.841	
Observations	5802		5297		5297		5297		5297	
Unique individuals	2236		2092		2092		2092		2092	

	**(6) Using social networking sites**		**(7) Reading news/ newspaper/ blog websites**		**(8) Streaming/ downloading TV/ radio, music, games, or ebooks**		**(9) Getting information about Government services**		**(10) Finding information on health-related issues**	
Women (ref: Wave 9)										
Wave 8	-0.043	(0.028)	0.040	(0.035)	-0.002	(0.036)	0.057	(0.036)		
COVID-19	-0.079***	(0.023)	-0.008	(0.025)	-0.145***	(0.028)	0.171***	(0.028)	-0.401***	(0.045)
Time varying covariates	Yes		Yes		Yes		Yes		Yes	
Mean outcome at Wave 9	0.609		0.498		0.611		0.180		0.778	
Observations	5970		5970		5970		5970		4441	
Unique individuals	2484		2484		2484		2484		2456	
Men (ref: Wave 9)										
Wave 8	-0.028	(0.022)	-0.045*	(0.027)	0.043	(0.036)	0.019	(0.029)		
COVID-19	-0.040**	(0.020)	-0.024	(0.020)	-0.114***	(0.029)	0.078***	(0.024)	-0.430***	(0.033)
Time varying covariates	Yes		Yes		Yes		Yes		Yes	
Mean outcome at Wave 9	0.514		0.636		0.628		0.309		0.831	
Observations	5639		5639		5639		5639		4182	
Unique individuals	2346		2346		2346		2346		2309	
Born by 1945 (ref: Wave 9)										
Wave 8	-0.019	(0.017)	0.025	(0.021)	0.072*	(0.037)	0.039*	(0.020)		
COVID-19	-0.077***	(0.018)	0.033	(0.020)	-0.117***	(0.033)	0.083***	(0.020)	-0.389***	(0.032)
Time varying covariates	Yes		Yes		Yes		Yes		Yes	
Mean outcome at Wave 9	0.316		0.381		0.455		0.138		0.724	
Observations	2139		2139		2139		2139		1465	
Unique individuals	865		865		865		865		830	

Born after 1945 (ref: Wave 9)										
Wave 8	-0.044	(0.036)	-0.036	(0.039)	-0.004	(0.039)	0.018	(0.043)		
COVID-19	-0.057**	(0.028)	-0.005	(0.027)	-0.125***	(0.030)	0.153***	(0.032)	-0.432***	(0.041)
Time varying covariates	Yes		Yes		Yes		Yes		Yes	
Mean outcome at Wave 9	0.615		0.603		0.654		0.264		0.820	
Observations	9471		9471		9471		9471		7158	
Unique individuals	3965		3965		3965		3965		3935	
No degree (ref: Wave 9)										
Wave 8	-0.046*	(0.024)	-0.009	(0.034)	0.049	(0.053)	0.031	(0.029)		
COVID-19	-0.067**	(0.027)	0.037	(0.026)	-0.123***	(0.045)	0.175***	(0.027)	-0.252***	(0.044)
Time varying covariates	Yes		Yes		Yes		Yes		Yes	
Mean outcome at Wave 9	0.530		0.382		0.511		0.102		0.623	
Observations	2590		2590		2590		2590		1902	
Unique individuals	1143		1143		1143		1143		1110	
With degree (ref: Wave 9)										
Wave 8	-0.030	(0.024)	-0.018	(0.026)	0.002	(0.026)	0.035	(0.030)		
COVID-19	-0.063***	(0.019)	-0.011	(0.019)	-0.122***	(0.020)	0.124***	(0.023)	-0.463***	(0.029)
Time varying covariates	Yes		Yes		Yes		Yes		Yes	
Mean outcome at Wave 9	0.573		0.616		0.650		0.281		0.854	
Observations	9017		9017		9017		9017		6721	
Unique individuals	3687		3687		3687		3687		3655	
Less wealthy (ref: Wave 9)										
Wave 8	-0.033	(0.024)	-0.006	(0.029)	0.053	(0.040)	0.038	(0.029)		
COVID-19	-0.089***	(0.021)	-0.010	(0.021)	-0.141***	(0.032)	0.162***	(0.024)	-0.369***	(0.036)
Time varying covariates	Yes		Yes		Yes		Yes		Yes	
Mean outcome at Wave 9	0.596		0.494		0.592		0.190		0.740	
Observations	6222		6222		6222		6222		4704	
Unique individuals	2687		2687		2687		2687		2637	
Wealthier (ref: Wave 9)										
Wave 8	-0.029	(0.025)	-0.018	(0.029)	-0.006	(0.029)	0.039	(0.033)		
COVID-19	-0.026	(0.021)	-0.003	(0.022)	-0.115***	(0.023)	0.086***	(0.027)	-0.449***	(0.032)
Time varying covariates	Yes		Yes		Yes		Yes		Yes	
Mean outcome at Wave 9	0.519		0.646		0.649		0.303		0.880	
Observations	5297		5297		5297		5297		3834	
Unique individuals	2092		2092		2092		2092		2078	

Notes: All are binary variables unless indicated otherwise. *p<0.10, **p<0.05, ***p<0.01. Figures in parentheses are standard errors. Following the specification in Figure 4 (full regression output in Table S7), time-varying covariates are age, partnership and employment status, perceived financial difficulties, self-reported health, depressive symptomology, loneliness, levels of positive and negative support from partner, and contact with others.

Table S9. Change in likelihood of daily Internet use, controlling for devices used to access the Internet and places where participants used the Internet.

	**(1) Interacted with type of device used**		**(2) Interacted with place where participants used the Internet**	
Wave (ref: Wave 9)				
Wave 8	0.011	(0.016)	0.021	(0.016)
COVID-19	-0.019*	(0.012)	-0.016	(0.013)
Interactions with place (ref: at home and elsewhere)				
At home only				
Wave 8			-0.008	(0.017)
COVID-19			0.036**	(0.016)
Elsewhere only				
Wave 8			-0.114**	(0.058)
COVID-19			-0.138*	(0.074)
Interactions with device (ref: smartphones and computers)				
Computers only				
Wave 8	-0.001	(0.016)		
COVID-19	0.013	(0.016)		
Smartphones only				
Wave 8	-0.095	(0.065)		
COVID-19	0.056	(0.047)		
Age	0.009	(0.006)	0.008	(0.007)
Married/cohabitating	-0.037	(0.036)	-0.021	(0.041)
Employment (ref: employed)				
Retired	0.014	(0.016)	-0.005	(0.017)
Other	-0.001	(0.018)	-0.022	(0.021)
Financial difficulties [a]	0.025	(0.019)	0.040*	(0.024)
Self-rated health [b]	0.025*	(0.013)	0.018	(0.014)
Has depressive symptomology [c]	0.008	(0.014)	0.019	(0.015)
UCLA Loneliness score [d]	0.004	(0.007)	-0.001	(0.007)
Positive support score [d]	-0.004	(0.015)	-0.018	(0.016)
Negative support score [d]	0.014**	(0.007)	0.011	(0.007)
Less than weekly contact with non-household members [e]	-0.016	(0.029)	-0.033	(0.029)
Mean outcome at Wave 9	0.845		0.843	
Observations	11718		8487	
Unique individuals	4475		3062	

Notes: All are binary variables unless indicated otherwise. *p<0.10, **p<0.05, ***p<0.01. Figures in parentheses are standard errors.

As the COVID-19 Substudy did not collect information on devices used to access the Internet or on places where they used the Internet, we take this information from Wave 9 (2018-19) and Wave 7 (2014-15), respectively. For the former analysis we categorise types of devices used into: (1) using smartphones and computers (defined as either, or any combination of, desktop, laptop, and tablet), 66% of sample; (2) only computers, 31%; or (3) only smartphones, 5%. In the latter we categorise places where they used the Internet into: (1) at home and elsewhere (work, education, another person's home, on the move, library, or internet café), 59% of sample; (2) only at home, 39%; or (3) only elsewhere, 2%.

There were no differences in changes in likelihood of daily Internet use since the outbreak, between participants who used both smartphones and computers (desktop, laptop, or tablet), those who used only smartphones, and those who used only computers, to access the Internet. Compared with participants who (as reported in 2014/15) used the Internet both at home and elsewhere (e.g., at work, education, another person's home, on the move, library, or internet café), those who used the Internet only elsewhere were indeed less likely to have increased to daily Internet use since the outbreak, but this was only a marginally significant difference; whereas those who used the Internet only at home were more likely to have increased to daily Internet use since the outbreak. However, neither of these three groups had a significant change in likelihood of daily Internet use since the outbreak, compared to the period prior to the outbreak.

Appendix references

1. Office for National Statistics. *Internet access – Households and individuals, Great Britain: 2020*. https://www.ons.gov.uk/peoplepopulationandcommunity/householdcharacteristics/homeinternetandsocialmediausage/bulletins/internetaccesshouseholdsandindividuals/2020 (2020).
2. Steptoe, A. & Fancourt, D. Leading a meaningful life at older ages and its relationship with social engagement, prosperity, health, biology, and time use. *Proc Natl Acad Sci U S A* **116**, 1207–1212 (2019).
3. di Gessa, G. & Price, D. Changes in health and social well-being in the COVID-19 clinically vulnerable older English population during the pandemic. *J Epidemiol Community Health* **75**, 1070–1077 (2021).
